# Non-rhizobial endophyte recruitment and diversity in *Pisum sativum* are strongly shaped by phosphorus fertilizer form

**DOI:** 10.1186/s40793-025-00751-0

**Published:** 2025-07-21

**Authors:** Stefanie Katharina Thaqi, Natalia Hensel, Nora Vitow, Christel Baum, Lisa-Marie Streb, Susanne Kublik, Peter Leinweber, Kerstin Panten, Michael Schloter, Stefanie Schulz

**Affiliations:** 1 Chair of Environmental Microbiology, TUM School of Life Sciences, Technische Universität München, Emil-Ramann-Straße 2, 85354 Freising, Germany; 2 Chair of Crop Physiology, TUM School of Life Sciences, Technische Universität München, Alte Akademie 12, 85354 Freising, Germany; 3 Research Unit Comparative Microbiome Analysis, Helmholtz Zentrum München, Ingolstädter Landstraße 1, 85764 Neuherberg, Germany; 4 Chair of Soil Science, University of Rostock, Justus-Von-Liebig-Weg 6, 18059 Rostock, Germany; 5 Federal Research Centre for Cultivated Plants, Institute for Crop and Soil Science, Julius Kühn Institute (JKI), Bundesallee 58, 38116 Brunswick, Germany

**Keywords:** Bone char, Non-rhizobial endophytes, Amplicon, Nodulation, *Pisum sativum*, Arbuscular mycorrhizal fungi, *nifH*

## Abstract

**Background:**

Non-rhizobial endophytes (NREs) support plant health and nodule function by enhancing symbiotic interactions and nitrogen fixation. However, their recruitment dynamics under fertilizers of varying phosphorus solubility remain poorly understood. This study investigated how four P fertilization treatments—no phosphorus (P0), bone char (BC), surface-modified bone char plus (BC^plus^), and triple superphosphate (TSP)—with increasing solubility influence microbial recruitment and diversity in *Pisum sativum*, leading to differences in plant-available phosphorus across bulk soil, rhizosphere, roots, and nodules.

**Results:**

Using 16S rRNA amplicon sequencing, we found that nodule-associated microbial communities were primarily recruited from unknown sources, likely seeds, followed by roots, especially under BC^plus^. Phosphorus solubility of treatments significantly influenced recruitment patterns, with solubility further shaping microbial diversity. BC^plus^ recruited beneficial taxa like *Beijerinckiaceae* and *Flavobacteriaceae*, which are associated with nitrogen fixation and biocontrol. In contrast, the highly soluble TSP treatment expanded recruitment from the rhizosphere, reflecting less stringent environmental filtering and promoting taxa like *Steroidobacteraceae* and *Blastocatellaceae*, known for nutrient cycling and pathogen suppression. In the absence of P fertilization (P0), recruitment relied heavily on seeds and roots, with arbuscular mycorrhizal fungi colonization prioritized over nodulation. Notably, TSP supported significantly more nodules with greater microbial diversity, potentially enhanced by NREs.

**Conclusions:**

Phosphorus solubility of the applied fertilizers strongly influences NRE recruitment dynamics in *P. sativum*. Seeds and roots act as primary reservoirs, while highly soluble fertilizers promote broader recruitment from the rhizosphere and increase microbial diversity in nodules. These results underscore the importance of the fertilization form in modulating NRE recruitment.

**Supplementary Information:**

The online version contains supplementary material available at 10.1186/s40793-025-00751-0.

## Background

In the last decades, pea (*Pisum sativum)* has gained more attention in agriculture due to its high nutritional value and its symbiotic interactions with microbes, which could improve soil quality. Its seeds are rich in protein (23–25%), slow-digesting starch, and other essential nutrients like vitamins and minerals [1, 2]. Beyond nutritional benefits, *P. sativum* plays a key role in crop rotations through symbiotic interactions with arbuscular mycorrhizal fungi (AMF) and diazotrophic rhizobia, enhancing nutrient cycling and soil health [3–5]. The mutualistic relationship between *P. sativum* and rhizobia is critical for nitrogen (N) fixation and nodulation with *Rhizobium leguminosarum* bv. *viciae* being the most commonly detected symbiont [6, 7].

While nodules were traditionally thought to host only rhizobia, recent studies show that they also harbor non-rhizobial endophytes (NREs), including genera such as *Agrobacterium*, *Burkholderia*, *Methylobacterium*, and *Ralstonia* [8]. NREs contribute to plant fitness by facilitating phosphorus (P) solubilization, indole-3-acetic acid (IAA) production, and pathogen suppression [9–12], although the full extent of their functional roles and interactions remain unclear [13]. While it is evident that nodules harbor diverse NREs, their recruitment pathways and precise roles in plant–microbe interactions still need to be fully understood. Mayhood and Mirza [9] observed a significant overlap between rhizosphere and nodule-associated bacteria, suggesting the rhizosphere as a potential source of NREs. Supporting this, it was demonstrated that most amplicon sequence variants (ASVs) of nodules originate from root-associated [14] microbes, which are strongly influenced by rhizosphere microbial communities. On top of the interactions between rhizosphere microbiome and rhizobia, AMF may also influence NREs. While AMF and rhizobia can compete for plant resources, potentially suppressing each other’s activity [15, 16], other studies highlight positive interactions that enhance N fixation and plant biomass [17–19]. Nonetheless, the role of AMF in shaping NRE communities remains largely unexplored.

It remains to be seen whether agricultural management practices influence NRE community structure. It is well understood that N and P availability plays a central role in nodule development and symbiotic N fixation. Nodules contain 2–3 times more P than other plant tissues, underscoring its importance in these processes [20–23]. Consequently, higher P levels have been linked to increased nodulation in legumes [24]. Highly soluble commercial P fertilizers have been shown to reduce diazotrophic diversity, potentially limiting successful nodulation. In contrast, slow-releasing fertilizers, such as biochar-based amendments, enhance diazotrophic abundance and diversity by altering community structure and promoting nutrient cycling [25]. This aligns with findings that long-term mineral fertilizer application decreases soil microbial abundance [26], whereas slow-releasing P fertilizers are associated with increased microbial abundance [27, 28], enhance diversity [29, 30], and promote greater activity in nutrient cycling [31].

Given *P. sativum*’s ability to establish symbioses with AMF and rhizobia, it is an ideal model plant to study how the tripartite interactions between AMF, rhizobia, and microbes at the plant-soil interface the composition and recruitment dynamics of NREs. In this study, we investigated how P fertilization with fertilizers of different solubility—control (P0) < bone char (BC) < surface-modified bone char plus (BC^plus^) < triple superphosphate (TSP)—affects microbial recruitment in bulk soil, rhizosphere, roots, and nodules during the ninth year of a long-term experiment. Despite identical P application rates across treatments, fertilizers with higher solubility resulted in increased plant-available P in soil over time [32]. By analyzing these interactions, we aimed to understand how fertilizer solubility, shaped by fertilizer form and solubility, influences microbial recruitment strategies and symbiotic relationships in pea-associated compartments.

To address these questions, we tested the following hypotheses: (i) Highly soluble P fertilizers reduce microbial diversity while shifting community composition in pea-associated compartments—particularly in roots and nodules—by favoring a few dominant taxa. (ii) NREs are primarily recruited from root and rhizosphere communities, with stronger recruitment occurring under highly soluble fertilizers, especially in the TSP treatment. (iii) P fertilization affects the balance between nodulation and AMF colonization, with low-solubility inputs favoring AMF and high-solubility inputs promoting nodulation and NRE diversity.

## Results

### Overall community responses to fertilization

**Fig. 1:**
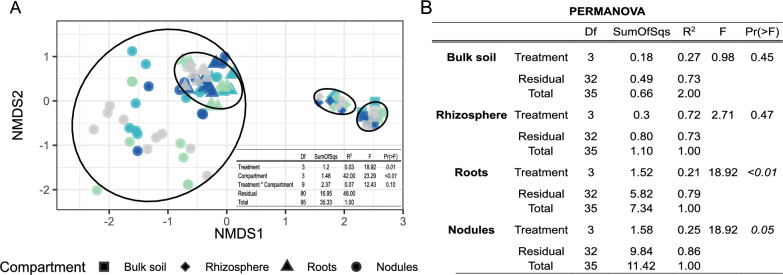
Impact of P fertilization on microbial composition across soil and plant compartments. **A** Non-metric multidimensional scaling (NMDS) plot of beta diversity based on Bray–Curtis dissimilarities. Samples are grouped by compartment with different shapes (bulk soil: square; rhizosphere: rhombus; roots: triangle; nodules: point) and by fertilization treatment with distinct colors (P0: grey, BC: turquoise, BC^plus^: light blue, TSP: blue). Ellipses represent 95% confidence regions for species clusters (stress = 0.17). Sample sizes: bulk soil (n = 12), rhizosphere (n = 12), roots (n = 36), nodules (n = 36). (B) PERMANOVA results based on Bray–Curtis dissimilarities. The table shows effect size (R^2^), significance (F-statistic), degrees of freedom (Df), and variance explained (SumOfSqs) for compartments and fertilization treatments. Globally, compartments explained 42% of the variation (*p* < 0.01), while fertilization treatment contributed 3% (*p* = 0.01). The interaction was not significant. Compartment-specific analysis showed the strongest fertilization effect in roots (R^2^ = 0.21, *p* < 0.01), followed by nodules (R^2^ = 0.14, *p* = 0.05), with minimal, non-significant effects in bulk soil and rhizosphere

Non-metric multidimensional scaling (NMDS) analysis revealed significant effects of compartment type and fertilization treatment on microbial community composition (Fig. 1A). Compartment type explained the majority of the variance (R^2^ = 0.42, *p* < 0.01), while fertilization treatment contributed marginally (R^2^ = 0.03, *p* = 0.01). A compartment-specific PERMANOVA analysis (Fig. 1B) showed minimal influence of fertilization in bulk soil (R^2^ = 0.27, *p* = 0.45) and the rhizosphere (R^2^ = 0.72, *p* = 0.47), while stronger effects were observed in roots (R^2^ = 0.21, *p* < 0.01) and nodules (R^2^ = 0.14, *p* = 0.05). Given these findings, subsequent analyses were conducted separately for each compartment to better understand the specific effects of fertilization within different microbial environments.

### Bulk soil and rhizosphere communities

**Fig. 2:**
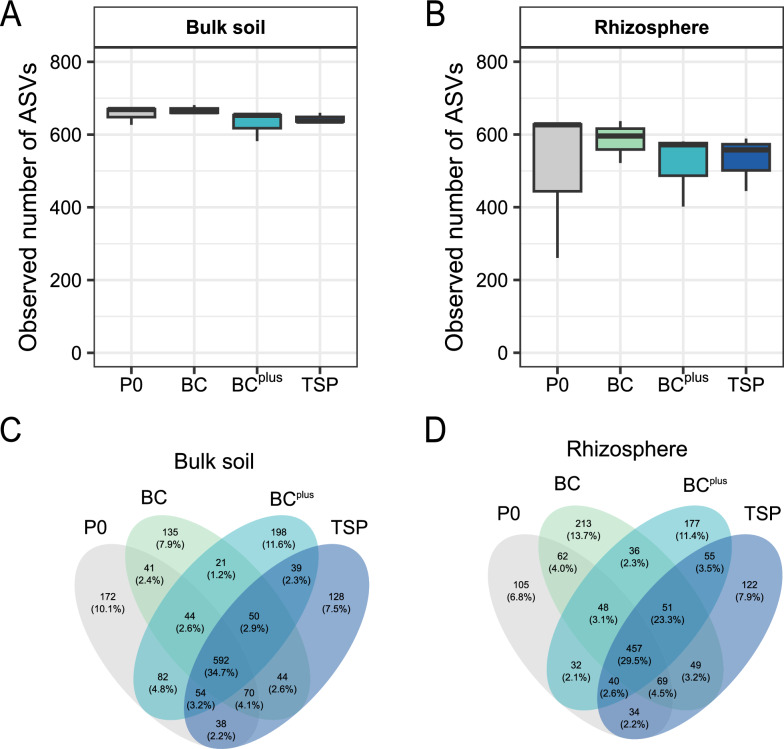
Microbial alpha diversity and shared ASVs in bulk soil and rhizosphere. Observed bacterial alpha diversity (ASVs) across P fertilization treatments (P0, BC, BC.^plus^, TSP) in **A** bulk soil and **B** rhizosphere (n = 3) is shown as box plots. No significant differences were detected among treatments (ANOVA, *p* > 0.05). Venn diagrams in (**C**) and (**D**) depict the number of shared and unique ASVs in bulk soil and rhizosphere, respectively. Values in brackets indicate the proportion of reads assigned to these ASVs. Additional information on the top 20 core and unique ASVs per treatment is provided in Supplementary Fig. S3

**Fig. 3:**
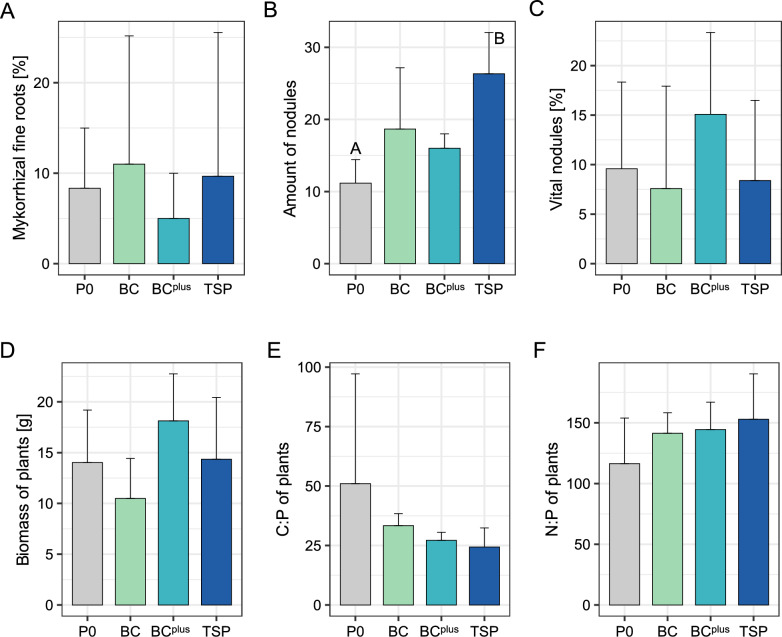
Plant properties under different P fertilization treatments. Plant properties were analyzed under four fertilization treatments with increasing phosphorus solubility: no P (P0), bone char (BC), surface-modified bone char (BC^plus^), and triple superphosphate (TSP). Parameters include: **A** percentage of mycorrhizal fine roots per plant (n = 3), **B** number of nodules per plant (n = 9), **C** percentage of vital nodules per plant (n = 9), **D** plant biomass (n = 3), **E** carbon-to-phosphorus (C:P) ratio (n = 3), and **F** nitrogen-to-phosphorus (N:P) ratio (n = 3). Data are presented as mean values with error bars representing standard deviation. Statistical differences between treatments were assessed using ANOVA with Tukey post-hoc tests. Significant differences between treatments (*p* < 0.05) are indicated by different capital letters. If no significant differences were detected, no letters are shown

Fertilization treatments did not significantly affect the number of observed ASVs in bulk soil (Fig. 2A) and rhizosphere samples (Fig. 2B). *Rhizobiaceae*, while more abundant in BC^plus^, remained low compared to endophytic compartments and were not significantly influenced by fertilization (Fig. S1). This trend was supported by *nifH* gene quantification and mirrored the increase in *Rhizobiaceae* under BC^plus^ (Fig. S2A). Conversely, AMF abundance and mycorrhizal colonization were highest in P0 and TSP, with BC^plus^ exhibiting the lowest levels, though these patterns were not statistically significant (Figs. 3A, S2B).

Only the C:P ratio of plant-available soil nutrients differed significantly, with BC (0.69) exceeding TSP (0.48; *p* = 0.05; Tables S1, S2). Other parameters, including plant biomass (Fig. 3D), total C:P (Fig. 3E) and N:P (Fig. 3F) ratios of plant material and N:P ratios of plant-available soil nutrients (Table S1), were unaffected by fertilization. P_CAL_ values, which reflect the concentration of plant-available P in the soil, increased progressively with the solubility of the applied fertilizers, despite identical P application rates (Table S1). This trend was accompanied by a slight increase in grain yield, ranging from 2.79 t ha^−1^ in P0 to 3.14 t ha^−1^ in TSP (unpublished data).

In bulk soil, 592 ASVs were shared across treatments (Fig. 2C), with dominant families including *Micrococcaceae, Gaiellaceae*, and *Oxalobacteraceae* (Fig. S3A). Most unique ASVs were observed for the BC^plus^, while P0 had the highest abundance of unique ASVs, including families like *Ktedonobacteraceae*, *Rhodanobacteraceae,* and *Caulobacteraceae* (Fig. S3B)*.*

In the rhizosphere, 457 ASVs were shared among all treatments, as shown in the Venn diagram (Fig. 2D). These shared ASVs were dominated by families such as *Rhizobiaceae*, *Burkholderiaceae*, and *Caulobacteraceae*, based on relative abundance patterns (Fig. S3A). Although the core community structure was relatively stable, shared ASVs exhibited treatment-dependent differences in their relative abundances, indicating a dynamic microbial response to fertilization (Fig S3A). In terms of unique ASVs, the highest number was observed under BC treatment, followed by BC^plus^ (Fig. 2D). The composition of unique ASVs, annotated at the family level, differed markedly across treatments (Fig. S3B). In TSP, they were dominated by *Morganellaceae, Xanthomonadaceae,* and *Sphingomonadaceae*, while BC was enriched in *Caulobacteraceae, Xanthomonadaceae,* and *Comamonadaceae*. In P0, *Ktedonobacteraceae* was the most abundant family, followed by *Opitutaceae* and *Xanthobacteraceae*. BC^plus^ unique ASVs included *Ktedonobacteraceae, Streptomycetaceae,* and *Burkholderiaceae*. Notably, some unique ASVs introduced new families, such as *Morganellaceae, Chitinophagaceae*, and *Sphingobacteriaceae*, absent in the top 20 core ASVs, highlighting distinct microbial recruitment patterns for each treatment.

### Root endophytes

**Fig. 4:**
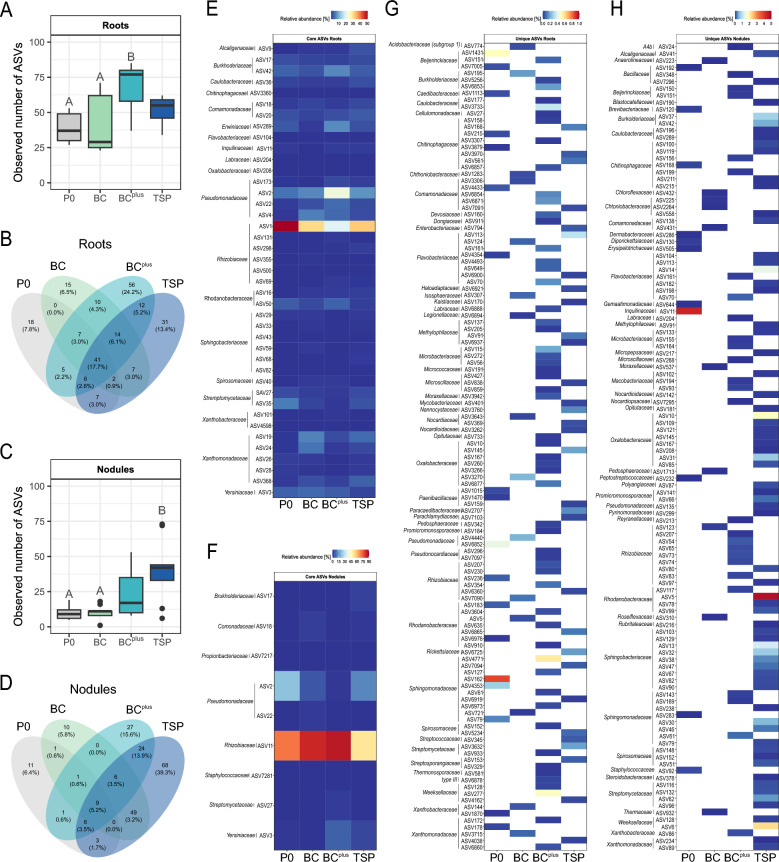
Microbial alpha diversity and distribution of shared and unique ASVs in roots and nodules under P fertilization treatments. Bacterial alpha diversity (observed ASVs) is shown for roots (n = 9) in panel (**A**) and for nodules (n = 9) in panel (**C**) as box plots. Significant differences between treatments were detected using ANOVA (p < 0.05) and are indicated by different capital letters; if no letters are shown, no significant differences were found. Venn diagrams in panels (**B**) and (**D**) illustrate the number of shared and unique ASVs across treatments (P0, BC, BC^plus^, TSP) in roots and nodules, respectively. Values in brackets indicate the proportion of reads assigned to the respective ASVs. Panels **E**–**H** present heatmaps showing the relative abundance of ASVs, with darker shades indicating higher abundance. Panel (**E**) displays core ASVs in roots shared across all treatments, panel **F** shows core ASVs in nodules, and panels **G** and **H** illustrate unique ASVs specific to individual treatments in roots and nodules, respectively

To validate sequencing accuracy in root samples, a mock community of eight known bacterial species was included as a positive control (Table S3). Seven out of eight species were successfully detected, with *Lactobacillus fermentum* missing, likely due to the high chloroplast DNA content. This result suggests that sequencing efficiency in root samples may be slightly influenced by plant-derived DNA. The number of observed ASVs in roots was significantly higher in the BC^plus^ compared to P0 and BC (*p* < 0.01; Fig. 4A). *Rhizobiaceae* dominated the root microbiome, with *Rhizobium phaseoli* being the abundant species in BC, followed by TSP, BC^plus^, and P0 (Figure S1). This trend corresponded with the lowest *nifH* gene copy numbers detected in P0 (Fig. S2A). Other *Rhizobiaceae* species were less abundant, with the highest diversity in P0 and TSP.

Among the ASVs shared acorss all treatments, two ASVs assigned to *Rhizobiaceae* and *Pseudomonadaceae* were predominant, with the latter showing the highest abundance in BC^plus^ (Fig. 4E). All other shared ASVs were present at low abundance across treatments. The root microbiome had a higher proportion of unique ASVs (Fig. 4B) compared to soil compartments (Fig. 2C, D). Within root samples, BC^plus^ exhibited the highest number of unique ASVs, followed by TSP and fewer in P0 and BC. The distribution and taxonomic identity of unique ASVs across treatments are visualized in Fig. 4G. Unique ASVs varied by treatment, reflecting differences in P fertilizer solubility but were generally composed of rare taxa. For instance, P0 was enriched in unique ASVs from *Sphingomonadaceae* and *Beijerinckiaceae*, BC^plus^ from *Sphingobacteriaceae* and *Weeksellaceae*, TSP from *Flavobacteriaceae*, and BC from *Oxalobacteraceae*. Despite treatment-specific differences, several families, including *Comamonadaceae, Flavobacteriaceae*, and *Rhizobiaceae*, were consistently represented among unique ASVs across treatments.

### Nodule communities

To validate sequencing accuracy in nodule and root samples, a mock community of eight known bacterial species was included as a positive control (Table S3). While seven out of eight species were detected in root samples, all eight were present in nodules, confirming a higher sequencing robustness for this compartment.

The total number of nodules was significantly lower in the P0 treatment compared to TSP (*p* < 0.01; Fig. 3B), while the proportion of vital nodules remained consistent across treatments (Fig. 3C). The observed number of ASVs in nodules was highest in TSP, significantly exceeding BC and P0 (*p* < 0.01, Fig. 4C). *Rhizobiaceae* dominated the nodule microbiome across all treatments, with *Rhizobium phaseoli* as the prevalent species (Fig. S1). Subsequent BLAST analysis confirmed these sequences as *Rhizobium leguminosarum* bv. *viciae*, a key symbiont of *Pisum sativum*. The highest relative abundance of *Rhizobiaceae* was observed in P0 and BC^plus^, while TSP and BC exhibited lower levels. This trend corresponded with *nifH* gene copy numbers, which were lowest in TSP (Fig. S2A).

Despite the small number of shared ASVs across treatments (9) (Fig. 4D), these ASVs were among the most abundant ones in nodules in contrast to other compartments, where shared ASVs represent a minority (Fig. 4F). These shared ASVs were predominated by ASVs assigned to *Rhizobiaceae* and *Pseudomonadaceae*, with the former being most abundant in BC and BC^plus^ and the latter in P0 and TSP. Other shared ASVs, including those from the families *Burkholderiaceae, Comamonadaceae, Propionibacteriaceae, Staphylococcaceae, Streptomycetaceae,* and *Yersiniaceae,* were consistently present but in low abundance.

The nodule microbiome also exhibited a high diversity of unique ASVs, with variation increasing alongside P fertilizer solubility (Fig. 3H). The TSP treatment showed the highest number of unique ASVs (68), including families such as *Rhodanobacteraceae*, *Weeksellaceae*, *Oxalobacteraceae*, *Sphingobacteriaceae*, *Flavobacteriaceae*, and *Burkholderiaceae*. In contrast, P0 nodules contained only 11 unique ASVs, among which one affiliated with *Inquilinaceae* was highly abundant. BC (10 unique ASVs) and BC^plus^ (27 unique ASVs) were characterized by a more stable microbial composition, with BC^plus^ showing the highest number of unique *Rhizobiaceae* ASVs. Both BC and BC^plus^ demonstrated stabilizing effects on the nodule microbiome, maintaining a consistent abundance of unique ASVs, unlike the more pronounced shifts observed under TSP and P0.

### Recruitment patterns of nodular microbiome

**Fig. 5:**
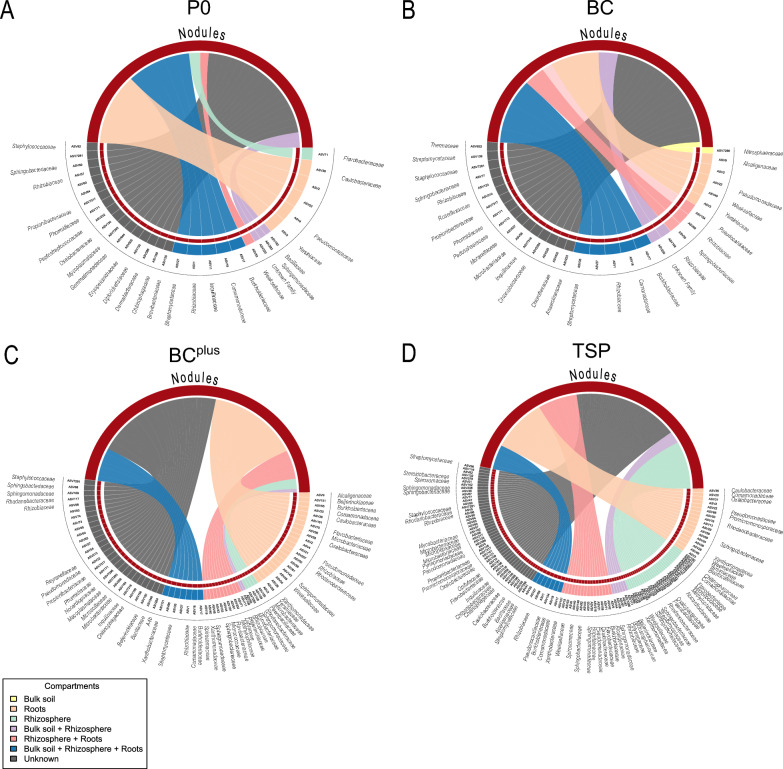
Sources contributing to the nodule-associated microbial community in *Pisum sativum* under P fertilization treatments. The chord diagram illustrates the proportional contributions of various microbial sources to the community composition in pea root nodules (sink represented in red) under four P fertilization treatments: **A** no P (P0), **B** bone char (BC), **C** surface-modified bone char (BC^plus^), and **D** triple superphosphate (TSP). The analyzed sources include Bulk soil (yellow), Rhizosphere (light green), Roots (orange), Bulk soil + Rhizosphere (lilac), Rhizosphere + Roots (light red), Bulk soil + Rhizosphere + Roots (blue), and Unknown (gray). Percentages represent the mean microbial contribution per treatment (n = 3). For P0, the majority of contributions originated from Unknown sources (42.64%) and Roots (24.51%), with smaller contributions from Rhizosphere + Roots (2.78%), Rhizosphere (3.70%), and Bulk soil + Rhizosphere + Roots (21.10%). Under BC treatment, Unknown sources (45.06%) and Roots (15.63%) contributed most, followed by Rhizosphere + Roots (10.23%) and Bulk soil + Rhizosphere + Roots (22.07%), with no contributions from the Rhizosphere alone. For BC.^plus^, contributions included Unknown sources (43.57%), Roots (30.17%), Rhizosphere + Roots (10.06%), Rhizosphere (3.54%), and Bulk soil + Rhizosphere + Roots (11.48%). Under TSP treatment, contributions shifted to Unknown sources (36.75%), Roots (19.13%), Rhizosphere + Roots (13.65%), Rhizosphere (17.30%), and Bulk soil + Rhizosphere + Roots (8.87%). Linear mixed-effects models followed by ANOVA on log-transformed data revealed significant differences between source compartments (*p* < 0.01; Table S2)

The sources of nodule-associated ASVs differed significantly across compartments (*p* < 0.01, Table S2). Overall recruitment patterns are visualized in Fig. 5. ASVs from unknown sources, potentially including seeds or unexplored reservoirs, contributed the most, followed by roots and ASVs shared between the rhizosphere and roots, which provided moderate contributions. The rhizosphere and bulk soil had lower contributions, with bulk soil consistently the least influential source. Pairwise comparisons confirmed significant differences between compartment contributions, with unknown sources contributing more ASVs than any other compartment and roots contributing more than the rhizosphere and bulk soil. Fertilization treatments alone did not significantly affect the number of ASVs recruited (Table S2). However, they influenced recruitment patterns within specific compartments (Fig. 5).

In the BC^plus^ treatment, 30.2% of ASVs found in nodules were also detected in roots, including members of *Beijerinckiaceae*, *Flavobacteriaceae*, *Microbacteriaceae*, and *Sphingomonadaceae*, as well as enriched *Pseudomonadaceae*. Nodules from TSP treatment shared 19.1% of their ASVs with roots, with additional contributions from *Alcaligenaceae*, *Comamonadaceae*, *Promicromonosporaceae*, *Rhodanobacteraceae*, *Spirosomaceae*, and *Streptomycetaceae*, and enrichment of *Sphingobacteriaceae* and *Xanthomonadaceae*. Nodules from BC and P0 treatments had similar proportions of root-derived ASVs (25.6% and 24.5%, respectively), largely affiliated with *Pseudomonadaceae*.

ASVs shared between the rhizosphere and roots contributed to nodule ASVs with varying proportions across treatments. Nodules in the TSP treatment recruited 13.7% of its nodule ASVs from this compartment, uniquely recruiting families such as *Chitinophagaceae, Methylophilaceae, Pseudomonadaceae, Rubritaleaceae*, and *Oxalobacteraceae*, alongside general enrichment of *Flavobacteriaceae* and *Sphingobacteriaceae.* Nodules from BC followed with 10.2%, recruiting ASVs primarily from *Rhizobiaceae,* while nodules from BC^plus^ recruited 10.1%, contributing unique families such as *Labraceae* and several ASVs from *Sphingobacteriaceae.* P0 showed the lowest recruitment (2.8%), with a single ASV recruited from the unique family *Weeksellaceae.*

Recruitment from the rhizosphere alone was highest under TSP (17.3%), significantly exceeding BC, where no ASVs were recruited from this compartment (*p* = 0.035). Nodules from TSP recruited ASVs from diverse families unique to this treatment, such as *Steroidobacteraceae, Blastocatellaceae,* and *Sphingomonadaceae,* while also generally enriching *Sphingomonadaceae* alongside other families. Nodules from BC^plus^ (3.5%) and P0 (3.7%) showed minimal contributions from the rhizosphere, with nodules from BC^plus^ recruiting ASVs from unique families like *Labraceae* and *Sphingobacteriaceae,* and P0 contributing a single ASV from the unique family *Weeksellaceae.*

Recruitment from ASVs shared between bulk soil and the rhizosphere was limited. In the TSP treatment, nodules recruited 4.3% of their ASVs uniquely from families such as *Oxalobacteraceae, Polyangiaceae,* and *Rhizobiaceae,* though no family was enriched. P0 nodules recruited a slightly higher proportion (5.3%) but with lower diversity and no unique family contributions. BC nodules recruited 6.3%, predominantly from *Rhizobiaceae,* without notable diversity, while BC^plus^ nodules recruited only 1.2%, showing minimal contributions from this shared compartment.

ASVs originating from bulk soil, rhizosphere, and roots were distributed across treatments. Nodules from BC recruited the highest proportion (22.1%) of ASVs from this combined compartment, primarily from *Rhizobiaceae.* P0 nodules followed closely with 21.1%, while BC^plus^ nodules recruited 11.5% and TSP nodules only 8.9%. *Rhizobiaceae* was consistently enriched under BC and TSP, reflecting its central role in nodule formation. Other families, such as *Burkholderiaceae* and *Streptomycetaceae,* were variably recruited across treatments without showing significant enrichment patterns.

ASVs from unknown sources, potentially including seed-associated microbiota, showed the highest recruitment proportions across treatments. BC nodules recruited 45.1% of its nodule ASVs from unknown sources, predominantly from *Rhizobiaceae,* with no other enriched families. Similarly, BC^plus^ nodules relied on unknown sources for 43.6% of its nodule ASVs, uniquely recruiting families like *A4b, Beijerinckiaceae, Microscillaceae,* and *Nocardiopsaceae,* with notable enrichment of *Rhizobiaceae.* Nodules from P0 derived 42.6% of its nodule ASVs from unknown sources, including unique families such as *Anaerolineaceae, Dermabacteraceae,* and *Peptostreptococcaceae,* though enrichment was again limited to *Rhizobiaceae.* In contrast, nodules from TSP relied least on unknown sources (36.8%) but uniquely recruited ASVs from a wide array of families, including *Burkholderiaceae, Caulobacteraceae, Flavobacteriaceae, Micropepsaceae, Opitutaceae, Oxalobacteraceae, Phormidiaceae, Pseudomonadaceae, Pyrinomonadaceae, Sphingobacteriaceae,* and *Streptomycetaceae,* with substantial enrichment of *Oxalobacteraceae, Sphingobacteriaceae, and Rhizobiaceae.*

## Discussion

This study demonstrates that P fertilizer solubility significantly impacts microbial communities in plant-associated compartments, with stronger effects observed in roots and nodules than in soil compartments. Importantly, we show that although all plots received the same total amount of P and started from soils classified as class C (the optimal range for plant-available P in the German system), differences in fertilizer solubility led to varying P_CAL_ levels over the course of the 9-year field experiment. At the time of sampling, P_CAL_ values ranged from just below the class C threshold in P0 to the upper end of the range in TSP, reflecting diverging trends in P availability driven by the solubility of the treatments. These differences clearly influenced microbial community structure and diversity. A progressive decrease in alpha diversity from bulk soil to nodules was observed, reflecting the selective pressures exerted by plant proximity. These findings underscore the importance of P fertilizer solubility as a key factor shaping plant–microbe interactions, particularly within symbiotic compartments. These findings are in line with previous studies showing that available P strongly shapes rhizosphere microbial diversity [33–36].

### Stability of microbial communities in bulk soil and rhizosphere

Bacterial richness and diversity in the bulk soil and rhizosphere remained remarkably stable across fertilization treatments, likely reflecting the buffering capacity of these compartments against fertilization-induced changes. This stability may be attributed to pre-existing soil nutrient levels, long-term adaptation of microbial communities, and the influence of factors such as organic C content and pH [37, 38]. Many ASVs were shared across fertilization treatments in bulk soil and rhizosphere, highlighting their role in maintaining stable and resilient microbial communities. These taxa likely play a key role in nutrient cycling and resilience to environmental fluctuations, supporting soil stability and adaptability under fertilization, as shown in previous studies [39–42]. Shifts in the relative abundance of shared ASVs suggest minor adjustments that help them function better under different nutrient conditions. In contrast, unique ASVs exhibited greater variability, highlighting their sensitivity to fertilization. For instance, the enrichment of families such as *Rhodanobacteriaceae* and *Ktedonobacteraceae* under P0 suggests adaptive responses to low P availability, including enhanced nutrient cycling mechanisms [43, 44]. Fertilization treatments like TSP and BC influenced the prevalence of specific taxa, with *Morganellaceae* abundant under TSP and *Caulobacteraceae* enriched in BC. The association of *Caulobacteraceae* with bone char-amended soils and its ability to utilize root-derived C may explain its prominence under BC, where steady P release supports nutrient cycling [45–47]. The abundance of *Morganellaceae* under TSP aligns with studies identifying this family in plant-associated compartments despite its common link to insect endosymbionts [48–50]. This suggests that plants may foster environments favorable to these microbes, potentially influencing interactions with arthropods indirectly.

### Solubility of P fertilization treatments influences diversity in roots

P fertilization significantly shaped root-associated microbial communities, with distinct responses to varying P solubility. Consistent with previous findings [51], root microbiomes showed sensitivity to nutrient availability, exhibiting noticeable shifts in bacterial composition and richness across treatments. Among root-associated ASVs, a small but abundant group shared across treatments demonstrates the selective pressures favoring essential functions, particularly in nutrient cycling, as exemplified by taxa such as *Rhizobiaceae* and *Pseudomonadaceae.* In low-P treatments (P0 and BC), unique ASVs like *Acidobacteriaceae* and *Chthoniobacteraceae* were enriched, reflecting adaptations to nutrient-limited conditions and their roles in nutrient cycling [52, 53]*.* P0 showed the highest abundance of unique ASVs, with taxa like *Sphingomonadaceae, Beijerinckiaceae,* and *Pseudomonadaceae.* These taxa promote plant growth, nutrient cycling, and stress resilience. For example, Pseudomonadaceae aids in IAA production and P solubilization [54], while *Beijerinckiaceae* contributes to P cycling, N fixation, and methane degradation, though it declines in high-P conditions [55–57]. *Sphingomonadaceae* enhances resilience to environmental stress through phytohormones and siderophores [58–61]. These adaptations suggest that P0-treated plants actively recruit microbial partners like *Pseudomonadaceae* and *Sphingomonadaceae* to enhance nutrient recycling and stress resilience under low P conditions.

In BC^plus^ and TSP, the higher solubility of the applied P fertilizers supported greater microbial diversity and unique ASVs, aligning with studies suggesting that elevated inorganic P availability and nutrient inputs can foster root microbial diversity and broader community shifts [62, 63]. Interestingly, while the total amount of unique ASVs was higher in BC^plus^, it was slightly reduced in TSP, aligning with the bell-shaped resource availability model [64], where diversity peaks at intermediate P levels and declines with excess nutrients. BC^plus^ supported taxa such as *Sphingobacteriaceae, Weeksellaceae*, and *Caulobacteraceae,* which thrive under steady nutrient release and more balanced P input. These taxa contribute to nutrient cycling and plant growth, with *Caulobacteraceae* playing a dual role in N cycling [47, 65] as well as promoting plant growth through siderophore production and supporting S oxidation [66–69]. This aligns with the properties of BC^plus^, which is enriched with reduced S compounds, promoting microbial sulfoxidation in the soil [70]. Meanwhile, *Weeksellaceae* contributes to N and P turnover, aiding nutrient balance [71, 72]. In contrast, the high solubility of TSP led to reduced microbial diversity but favored *Flavobacteriaceae,* a family known for its strong P-solubilizing capacity [73, 74] and plant health-promoting traits. These may synergize with *Rhizobiaceae* in supporting nodulation and nitrogen fixation.

### Different assembly of nodule microbiomes based on P fertilizer solubility

The balanced nodulation hypothesis suggests that legumes optimize N acquisition by maintaining dominant, efficient strains alongside low-density microbial diversity to balance C costs and symbiotic benefits. [75]. Our findings support this, showing that P fertilizer solubility shapes nodule-associated communities. Under TSP, significantly higher richness suggests broader recruitment of non-rhizobial endophytes, complementing symbiotic functions and reducing reliance on C-costly rhizobial symbioses, while dominance of *Rhizobiaceae* in the other treatments highlights a focus on efficient N fixation under lower P availability. This reflects legumes’ adaptive recruitment strategies to balance C costs and benefits.

This aligns with research highlighting the influence of soil properties [76] and fertilization on microbial communities, with studies indicating that P deficiency reduces nodule populations [77, 78].

High P availability has been shown to enable plants to allocate more C to a broader range of microbial partners, increasing diversity while reducing selectivity [79] Consistently, under TSP, nodules displayed the highest microbial diversity, including unique ASVs of families such as *Rhodanobacteriaceae* and *Sphingobacteriaceae,* which contribute to pathogen suppression and bioactive compound production [80, 81]. *Oxalobacteraceae,* exclusively found in TSP, is associated with enhanced microbial diversity and beneficial functions [82–84].

Conversely, P-limited conditions (P0) supported lower microbial diversity, dominated by ASVs of *Inquilinaceae* and *Rhizobiaceae. Inquilinaceae* contributes to nodulation-independent N fixation [85, 86]. *Rhizobiaceae* reflects the selective recruitment of highly efficient symbionts to minimize C costs under nutrient scarcity. This selective recruitment aligns with findings that plant species richness is associated with the recruitment of unique ASVs [40], and that P-limited plants may adjust their N acquisition strategies, favoring less C-intensive sources such as nitrate and ammonium [79].

The BC and BC^plus^ supported a balance between functional diversity and symbiotic stability in nodules. *Rhizobiaceae* dominated unique ASVs, emphasizing its central role in N fixation and symbiotic equilibrium, while low-abundance taxa, including plant growth-promoting microbes, likely contributed significant ecological functions [87].

Across treatments, a minimal set of shared ASVs accounted for a significant proportion of the relative abundance in nodules. *Rhizobiaceae* and *Pseudomonadaceae* were the main contributors to this group. *Rhizobiaceae* abundance was lowest in TSP, where highly soluble P was rapidly available, while *Pseudomonadaceae* showed the highest abundance under these conditions. Conversely, in the other treatments, *Rhizobiaceae* was more dominant, reflecting a preference for environments with lower or more gradually available P. In contrast, the abundance of *Pseudomonadaceae* was reduced. This inverse relationship aligns with previous findings reporting a negative correlation between Pseudomonas and Rhizobium [88].

### P fertilizer solubility influences the recruitment of the nodular community

The primary source of nodule-associated microbiota likely includes seed-associated microbes, highlighting the role of vertical transmission during early plant colonization [89–91]. These communities, shaped by the environment of the preceding plant generation, provide a stable foundation for further recruitment, particularly during early growth stages [92, 93]. In contrast, bulk soil contributed least across all treatments, suggesting a spatial filtering process from distal to proximal compartments.

Fertilization shaped microbial recruitment from roots and rhizosphere, primarily through its effect on plant-available P. Although P_CAL_ values did not differ significantly, a trend toward higher plant-available P with increasing fertilizer solubility was evident. This likely reflects both long-term microbial contributions to P mobilization and the high P demand of *P. sativum*, which may mask sharper treatment effects. Supporting this, earlier studies on the same field with different crops like winter barley, winter oilseed rape and winter wheat showed significantly higher plant-available P under more soluble fertilizers applied at identical rates [32, 94].

Roots emerged as key microbial reservoirs, especially under BC^plus^, where the moderately soluble P fertilizer promoted strong root-associated recruitment and diversity, likely supporting microbial coexistence and redundancy. These findings align with recent evidence highlighting the root as a central filter and assembly hub for the nodule microbiome [14]. Unique recruitment under BC^plus^ included families such as *Beijerinckiaceae* and *Flavobacteriaceae*—the former includes free-living nitrogen fixers involved in carbon and phosphorus cycling [57, 95, 96], while the latter is associated with biocontrol and plant health promotion [97]. In contrast, TSP recruited fewer microbes from roots but uniquely enriched beneficial families like *Alcaligenaceae, Rhodanobacteraceae,* and *Streptomycetaceae*, associated with nutrient cycling, pathogen suppression, and plant stress resilience [98–104]. BC and P0, representing low-solubility and no-P fertilization treatments, respectively, primarily recruited core taxa related to nitrogen fixation and plant health [6, 7, 54].

The rhizosphere contributed markedly under TSP, enriching taxa such as *Blastocatellaceae*, *Steroidobacteraceae*, and *Sphingobacteriaceae*, which are implicated in N cycle stabilization, nutrient retention, and disease suppression [105–111]. In contrast, rhizosphere-derived contributions were minimal under BC, BC^plus^, and P0, suggesting that rhizosphere-to-nodule recruitment is strongly influenced by P solubility. Enhanced recruitment under TSP likely reflects reduced environmental filtering, as the high solubility of the applied P fertilizer promotes increased root exudation and alleviates metabolic constraints [112]. These conditions may create broader ecological niches and promote microbial colonization. Long-term data from the same site further support this interpretation, as soluble fertilizers like TSP led to significant increases in P_CAL_ values over time, despite equal P inputs [32].

Microbial migration from bulk soil to nodules follows a stepwise path via rhizosphere and roots, shaped by both microbial traits and host filtering. Plants attract microbes via root exudates [113] with chemotaxis playing a key role [114]. AMF can facilitate this process by extending hyphal networks into the bulk soil, acting as “hyphal highways” that support bacterial dispersal and colonization [115–117]. In our study, elevated AMF colonization under P0 coincided with higher NRE diversity compared to BC, suggesting AMF may enhance NRE recruitment by supporting transport and modulating rhizosphere conditions. Prior research has shown that *Bradyrhizobium* and *Sinorhizobium* can migrate along AMF hyphae [115, 118], and AMF structures have been observed in aged nodules [119], indicating possible integration with the nodule microbiome. AMF also modulate P availability and root physiology [120], influencing exudate patterns and microbial assembly. Additional mechanisms include hitchhiking of non-motile bacteria by motile species like *Pseudomonas fluorescens* [121]. Interestingly, *Pseudomonadaceae* were frequently recruited from roots under P0, where AMF colonization was strongly enhanced. This pattern may reflect potential interactions with AMF, suggesting that AMF networks not only facilitate nutrient acquisition but may also promote microbial transport and colonization under P-limited conditions. Upon arrival at the rhizoplane, colonization typically begins with attachment via pili and extracellular polymeric substances [122], followed by surface colonization or endophytic entry through epidermal cracks or cell junctions [123–125]. *Rhizobiaceae* typically invade through curled root hairs and form infection threads, though “crack entry” pathways have also been described [126–131]. NREs are presumed to access nodules through similar mechanisms, though their exact entry routes remain less well defined [8, 132–136]. These mechanistic insights are reflected in our source-tracking results: while ASVs from all compartments were detected in nodules, root-derived ASVs consistently dominated, supporting a selective bottleneck at the root–nodule interface. For instance, *Pseudomonadaceae* were frequently recruited from both roots and rhizosphere under BC^plus^ and TSP; *Sphingobacteriaceae* were enriched primarily under TSP; and *Rhizobiaceae* were detected across all treatments, reflecting their central symbiotic role. Overall, fertilizer solubility modulated microbial diversity and transmission routes, with higher solubility promoting broader recruitment. While this may benefit plant growth, it could also increase the risk of opportunistic colonizers, indicating a potential trade-off between enhanced microbial diversity and host resilience [137, 138].

### P fertilizer solubility modulates symbiotic interactions

The solubility of P fertilizers plays a pivotal role in shaping symbiotic interactions in *P. sativum*, particularly the balance between nodulation and AMF colonization. Taxonomic classification based on the SILVA 16S rRNA database initially assigned the dominant ASVs to *Rhizobium phaseoli*. However, subsequent BLAST analysis identified these sequences as *Rhizobium leguminosarum* bv. *viciae*, the primary symbiont of *P. sativum*, confirming a stable host–symbiont relationship independent of P levels [6, 7].

Despite this taxonomic stability, P solubility significantly influenced community structure and symbiotic dynamics. High solubility in the TSP treatment led to the highest number of nodules, likely reflecting increased energy and nutrient availability that facilitates nodulation [139]. However, paradoxically, the abundance of *nifH*-harboring bacteria and rhizobia decreased under TSP, suggesting a shift toward NREs. These NREs may play a complementary role in enhancing nodulation [13, 140] or indirectly supporting plant growth through mineral supply and the synthesis of bioactive compounds [141]. AMF abundance remained remained pronounced, indicating potential synergistic interactions among AMF, rhizobia, and the host plant [142]. Under moderately soluble P fertilization, as in the BC^plus^ treatment, rhizobial symbiosis was favored over AMF, suggesting a functional prioritization of N fixation when nutrient levels are sufficient but not excessive. Conversely, the P0 treatment—where P_CAL_ values were lowest (Table S1) —AMF colonization was strongly enhanced, likely reflecting compensatory scavenging under low P conditions. This was accompanied by reduced nodulation and lower *nifH* gene abundance, further underscoring AMF’s role in P acquisition under nutrient limitation [143]. In the BC treatment, characterized by low P solubility, both nodulation and AMF colonization were reduced, likely reflecting a threshold below which neither symbiosis is energetically favorable. These findings highlight that the form and solubility of P fertilization not only affect nutrient availability, but also plays a key role in shaping which microbes establish themselves and how the plant manages its symbiotic partnerships to access nutrients.

## Conclusions

This study underscores the significant role of the form of P fertilizer in shaping microbial communities in pea-associated compartments, particularly in roots and nodules. The solubility of P had a major effect on diversity, recruitment, and function. The intermediate solubility of BC^plus^ encouraged diverse microbial communities and supported N fixation by balancing competition and coexistence. In contrast, the high P solubility of TSP increased recruitment from the rhizosphere, bringing in microbes linked to nutrient cycling and pathogen suppression, and may have supported a tripartite symbiosis among AMF, rhizobia, and NREs. However, TSP might also raise the risk of pathogen recruitment. Under low P conditions (P0), AMF colonization took priority over nodulation, reflecting adaptations to nutrient scarcity. Roots and the rhizosphere acted as primary sources for nodule-associated microbes, while bulk soil had a minor role. Notably, unknown sources, such as seed-associated microbiota, were also important, highlighting the need to study seeds in future research. These findings offer practical guidance for fertilization strategies that improve nutrient use and support sustainable farming.

## Methods

### Experimental setup and sampling

The field trial, established in 2014 in Braunschweig, Germany (52° 18′ N 10° 27′ E), was conducted on Dystric Cambisol and Haplic Luvisol [144] and is built from sandy fluviatil sediments overlaid with sandy loess. The soil pH averaged across all treatments during sampling was 6.1. The area receives an average annual precipitation of 620 mm and a mean temperature of 9.0 °C. The randomized block experiment included three replicates of four treatments: a non-P-fertilized control (P0), BC, BC^plus^ (S-amended bone char from biogas purification, patent DE102011010525), and TSP. TSP is highly soluble, BC^plus^ has intermediate solubility due to surface modification, and BC is a low-solubility P source [145]. Plots measured 5.75 m × 17.50 m, with P fertilizers applied annually at 45 kg ha^−1^ (reduced to 30 kg ha^−1^ since 2021) shortly before sowing. Over the years, despite equal P application rates, the fertilizers resulted in significant differences in P_CAL_ values due to their varying solubility, as demonstrated in previous analyses on the same field site [32, 94]. A 5-year crop rotation included winter barley, winter oilseed rape, winter wheat, lupin, and winter rye, with lupin replaced by *P. sativum* (cultivar Salamanca) in the second rotation cycle. Fields were plowed (25 cm), and the remaining straw was incorporated after harvest. Sampling occurred in June 2022, during the flowering stage (BBCH 68/69), when nodule biomass peaks [146–148].

Four compartments were sampled: bulk soil from pooled soil cores, rhizosphere soil by brushing roots, and root endosphere and nodules from surface-sterilized plant material. Bulk soil was pooled from 15 cores (10 cm depth) per plot and sieved to 2 mm. Rhizosphere soil also sieved to 2 mm, was collected by brushing the roots of two neighboring plants, which were also used for root and nodule sampling. Two neighboring plants per plot were used for analyses to increase the amount of sample material. All rhizosphere soil from these plants was collected, while all nodules and roots were harvested and subsequently surface-sterilized. Recognizing the high variability expected in endophytic samples, three extractions were performed for both roots and nodules per plot for molecular analyses. Roots and nodules were surface-sterilized [149]. Moreover, sterility was confirmed by performing a 35-cycle PCR with the remaining washing water and incubating a surface-sterile sample overnight on an R2A agar plate. All samples designated for molecular analysis were subsequently frozen at − 80 °C. Additionally, bulk soil samples were stored at 4 °C for soil chemical analysis, and three different plants per plot were sampled to assess nodulation and mycorrhization rates, with these samples also stored at 4 °C.

### Analysis of the plant and bulk soil material

Aboveground plant biomass was dried at 60 °C to constant weight in a ventilated oven to determine dry matter (DM). Dried samples were ground < 0.5 mm with an ultracentrifugal mill (Retsch ZM 200, Haan, Germany). N and C contents were analyzed using 30 mg (DM) material in an Elemental Analyzer (Euro EA, Eurovector, Italy). Plant P content was determined by digesting 0.1 g DM with 5 mL HNO_3_ and 3 mL H_2_O_2_ in a microwave (Mars Xpress, CEM, Kamp-Lintfort, Germany), diluting the digest to 25 mL with distilled H_2_O, and measuring P concentrations at 214.915 nm using ICP-OES (Optima 8300, Perkin Elmer, Waltham, Massachusetts, USA). Dissolved organic carbon (DOC) and total N bound (TN_b_) were extracted (1:20 soil: 0.01 M CaCl_2_) [150, 151] and quantified using DIMA-TOC 2000 + DIMA-N (Dima Tec, Langenhagen, Germany) or a Skalar (Skalar Analytical B.V., Breda, Netherlands), respectively. P_CAL_ was extracted with Calcium acetate lactate [152]. The extracts were quantified using ICP-OES at 213.6 nm (iCAP, Thermo Fisher, Cambridge, United Kingdom). Mycorrhizal colonization of fine roots was quantified using the intersection method [153] after treating 10 mm root segments with 10% KOH for 24 h, acidifying with 1% HCl for 15 min, and staining with 0.05% chlorazol black E for 24 h [154]. Nodules were categorized as active (red-pink, containing leghemoglobin) or inactive (white or grey) based on visual inspection [155].

### DNA extraction, qPCR, and 16S rRNA gene amplicon sequencing

DNA was extracted from 0.3 g of bulk or rhizosphere soil, 0.5 g of root, or one vital nodule using Lysing Matrix Tubes E (MP Biomedicals, USA) with the Precellys24 Instrument (Bertin Technologies, France). Nodules were sliced open before extraction, and roots were frozen in liquid nitrogen and crushed in a sterile mortar. DNA was extracted using a phenol–chloroform protocol [156, 157] and stored at − 20 °C. Extraction blanks served as negative controls. DNA quality and quantity were assessed via Nanodrop ND-1000 (Thermo Fischer Scientific, Ma, USA) and Quant-IT™ Pico-Green® dsDNA Assay Kit (Thermo Fischer Scientific, MA, USA), respectively.

Real-time quantitative PCR was performed in the 7300 Real-Time PCR System Machine (Applied Biosystems, Germany). qPCRs were conducted in 25 μL reactions containing 12.5 μL SYBR Green® Thermo Fisher Scientific, USA), forward and reverse primers (Metabion, Germany), 0.5 μL BSA (3%, Sigma, Germany), and DEPC-treated water. All reactions were run for 40 cycles, with optimal sample dilutions of 1:16 to 1:64. The size of randomly selected qPCR products was verified using a 1.5% agarose gel. The markers, primers, and thermal profiles are summarized in Table S4. Each run included standard curves (R^2^ > 0.99), no-template controls, and diluted samples. Efficiencies ranged from 78–108%, and samples with < 10 copies μl^−1^ were excluded.

For 16S rRNA gene amplicon sequencing, the V4 region was amplified using primers 515F (5′-GTGYCAGCMGCCGCGGTAA-3′) and 806R (5′-GGACTACNVGGGTWTCTAAT-3′) [158, 159]. PCR was performed with the Nextera® XT Index Kit v2 (Illumina) using NebNext® High-Fidelity 2X PCR Master Mix (New England Biolabs, Ipswich, MA, USA), 3% bovine serum albumin (BSA) (Sigma-Aldrich), and 5 ng/μL DNA. The following cycling conditions were used: 98 °C for 1 min, 35 cycles of 98 °C for 10 s, 55 °C for 30 s, 72 °C for 30 s, followed by a final extension at 72 °C for 5 min. A PCR negative control was included in each run to check for contamination. To account for potential sequencing biases, a mock community (ZymoBIOMICS™ Microbial) was included as a positive control for the nodule and root samples, where plant DNA is present, in accordance with the manufacturer’s protocol (Table S3). Before sequencing, all samples, including PCR negative controls, positive controls, and extraction blanks, were amplified and confirmed on a 1.5% agarose gel. Subsequently, PCR clean-up was carried out using Agencourt AMPure XP magnetic beads from Beckman Coulter Life Sciences (Brea, CA, United States), following the manufacturer’s protocol with a DNA-to-bead ratio of 0.8. DNA concentration and fragment size were assessed using the Fragment Analyzer™ (Agilent Technologies, Santa Clara, CA, USA). For indexing, 10 ng of DNA was used, and sample-specific indices were added using the Nextera® XT Index Kit v2 (Illumina). After a second clean-up, DNA concentration and quality were re-verified, and the final DNA was pooled, standardized to 4 nM, and sequenced on the Illumina MiSeq platform (2 × 300 bp, paired-end, 20% PhiX).

### Bioinformatic and statistical data analysis

Demultiplexed fastQ files generated by MiSeq were were pre-processed on the European Galaxy server [160]. Sequencing adapters were removed using AdapterRemoval v2 [161], and reads were trimmed (240 bp forward, 180 bp reverse) and denoised using DADA2 [162]. Taxonomy was assigned with the SILVA database version 138 [163]. For root and nodule samples, resequenced data were merged using the mergeSequenceTables function in DADA2, and ASV counts were summed across datasets to standardize the format. Microbiome analysis was performed in R (version 4.3.1) [164], with ‘decontam’ (version 1.13.0) used for contaminant removal [165] and ‘phyloseq’ for downstream analyses (version 1.44.0) [166]. Sequencing yielded 832,694 reads (bulk soil), 798,878 (rhizosphere), 5,160,695 (nodules), and 3,423,777 (roots). After filtering for contaminants, chloroplasts, and mitochondria, 550,081 reads remained for bulk soil, 615,179 for rhizosphere, 3,844,636 for nodules, and 605,857 for roots (Table S5). A mock community confirmed sequencing accuracy (Table S3). Cumulative sum scale (CSS) normalization [167] addressed sampling depth differences. For robust analysis of the endophytes, only ASVs present in at least two of three replicates per plot were retained [40]. Beta diversity was assessed using Bray–Curtis distances, visualized with NMDS plots, and tested for significance using PERMANOVA (adonis function, vegan; version 2.6–4; [168]). Venn diagrams (‘ggvenn’; version 0.1.10; [169]) and heatmaps (‘ggplot2’; version 3.3.5; [170] were used to illustrate unique and shared ASVs. Source tracking analysis quantified the contributions of compartments to nodular ASVs. Venn diagrams identified ASVs shared between nodules and other compartments. The percentage of nodular ASVs simultaneously present in bulk soil, rhizosphere, roots, or multiple compartments was calculated per block, averaged across treatments, and visualized using chord diagrams (‘circalize’; version 0.1.10; [171]).

Log-transformed data were analyzed with linear mixed-effect models (‘nlme’; version 3.1-162; [172]), and significant differences between treatments (*p* < 0.05) were assessed using 2-way ANOVA with Tukey post-hoc tests.

## Supplementary Information

Supplementary material 1.

## Data Availability

The amplicon sequences on 16S rRNA genes have been deposited in the Sequence Read Archive (SRA) of The National Center for Biotechnology Information (NCBI) (https://www.ncbi.nlm.nih.gov/sra) under the BioProject ID:PRJNA1128047.
