# Supplementary material for: Non-rhizobial endophyte recruitment and diversity in Pisum sativum are strongly shaped by phosphorus fertilizer form
Source: Environ Microbiome. 2025 Jul 21;20:92. doi: 10.1186/s40793-025-00751-0 (PMC12281753; doi:10.1186/s40793-025-00751-0)
Supplement: Supplementary file 1 — Supplementary material 1. [file 40793_2025_751_MOESM1_ESM.pdf]

Additional File 1

Table S1 Plant and soil parameters 2
Table S2 ANOVA results from linear mixed-effects models based on log-transformed data.......... 4
Table S3 Normalized proportions of positive controls in nodules and roots by treatment.......... 9
Figure S1 Relative abundance (%) of Rhizobiaceae 5
Figure S2 Quantification of gene copy numbers using qPCR 6
Figure S3 Heatmap showing the relative abundance of the top 20 unique and shared ASVs 7
Table S4.Primer, reaction mixture, conditions, and calibration standards for real-time qPCR.......... 8
Table S5 Read loss per processing step during the bioinformatic pipeline. 9

Table S1 Plant and soil parameters. Mean values and standard deviations (Mean ± SD) of total carbon (C), nitrogen (N), and phosphorus (P) concentrations in plant tissue, dissolved organic carbon (DOC) in bulk soil, total nitrogen bound (TN_b) in bulk soil, plant-available phosphorus (P_{cal}) in bulk soil, plant-available soil nutrient ratios (C:P, N:P) across treatments.

**Treatment**	**Total C [mg g^{-1}]**		**Total N [mg g^{-1}]**		**Total P [mg g^{-1}]**		**DOC [mg kg^{-1}]**		**TN_b in [mg kg^{-1}]**		**P_{cal} [mg kg^{-1}]**		**C:P in soil**		**N:P in soil**	
	mean	sd	mean	sd	mean	sd	mean	sd	mean	sd	mean	sd	mean	sd	mean	sd
P0	372.62	87.16	27.24	7.35	0.88	0.58	23.06	4.82	4.82	2.22	30.93	5.22	0.77	0.16	0.16	0.08
BC	401.52	9.50.	24.90	4.70	0.77	0.24	30.21	7.91	5.13	0.16	31.87	2.74	0.93	0.18	0.16	0.01
BC^{plus}	408.63	4.78	27.53	5.53	1.03	0.23	22.46	1.41	4.70	2.11	33.40	2.62	0.68	0.07	0.14	0.07
TSP	406.87	5.31	23.33	4.01	1.09	0.52	25.81	1.55	5.90	0.42	54.00	6.08	0.47	0.03	0.16	0.01

Table S2 ANOVA results from linear mixed-effects models based on log-transformed data. Significant values ($p < 0.05$) are highlighted in italics.

**Compartment**	***p-value***	**variable**
Bulk soil	0.13	AMF
Rhizosphere	0.07	
Bulk soil	0.27	*nifH*
Rhizosphere	0.87	
Nodules	0.06	
Roots	0.08	
Bulk soil	*0.05*	C:P in soil
Bulk soil	0.58	N:P in soil
Bulk soil	0.42	DOC
Bulk soil	0.71	TN_b
Bulk soil	0.67	P_{cal}
Plant	0.11	Biomass of plants
Plant	*<0.01*	Amount of nodules
Plant	0.48	Vitality of nodules
Plant	0.87	Mycorrhizal fine roots
Plant	0.12	C:P in plant
Plant	0.17	N:P in plant
Plant	0. 47	Total C in plant
Plant	0.67	Total N in plant
Plant	0.57	Total P in plant
Bulk Soil	0.20	Observed ASVs
Rhizosphere	0.83	
Roots	*<0.01*	
Nodules	*0.01*	
Treatment	0.99	Contribution to nodule-associated ASVs
Compartment	*<0.01*	
Treatment: compartment	0.27	

Table S3 Normalized proportions of positive controls in nodules and roots by treatment. Normalized proportions of the positive controls, including the mock community (ZymoBIOMICS®), compared to the relative abundance of these bacterial species in the nodules per treatment P0, TSP, BC, and BC^{plus}.

		**Nodules**		**Roots**		
	Theoretical [%]	Normalized proportion [%]	Relative abundance [%]	Normalized proportion [%]	Relative abundance [%]	
Pseudomonas aeruginosa	4.20%	4.02%	5.39%	4.86%	5.98%	P0
Escherichia coli	10.10%	8.56%	11.49%	12.22%	15.03%	
Salmonella enterica	10.40%	8.29%	11.13%	1.75%	2.15%	
Lactobacillus fermentum	18.40%	12.29%	16.50%	0.00%	0.00%	
Enterococcus faecalis	9.90%	6.04%	8.11%	9.12%	11.22%	
Staphylococcus aureus	15.50%	8.79%	11.81%	13.44%	16.53%	
Listeria monocytogenes	14.10%	9.65%	12.95%	15.01%	18.47%	
Bacillus subtilis	17.40%	15.80%	21.21%	22.16%	27.25%	
Pseudomonas aeruginosa	4.20%	2.77%	4.03%	4.33%	5.44%	BC
Escherichia coli	10.10%	6.90%	10.03%	10.17%	12.79%	
Salmonella enterica	10.40%	6.60%	9.59%	1.34%	1.68%	
Lactobacillus fermentum	18.40%	11.11%	16.15%	0.00%	0.00%	
Enterococcus faecalis	9.90%	5.11%	7.43%	7.45%	9.37%	
Staphylococcus aureus	15.50%	6.07%	8.82%	12.56%	15.80%	
Listeria monocytogenes	14.10%	8.19%	11.90%	12.95%	16.29%	
Bacillus subtilis	17.40%	12.63%	18.35%	17.65%	22.20%	
Pseudomonas aeruginosa	4.20%	3.01%	4.41%	3.44%	4.22%	BC^{plus}
Escherichia coli	10.10%	6.74%	9.88%	7.22%	8.85%	
Salmonella enterica	10.40%	6.44%	9.43%	1.13%	1.39%	
Lactobacillus fermentum	18.40%	11.60%	16.99%	0.00%	0.00%	
Enterococcus faecalis	9.90%	5.55%	8.13%	6.04%	7.40%	
Staphylococcus aureus	15.50%	6.96%	10.19%	9.62%	11.79%	
Listeria monocytogenes	14.10%	8.30%	12.16%	10.42%	12.76%	
Bacillus subtilis	17.40%	0.00%	0.00%	14.85%	18.19%	
Pseudomonas aeruginosa	4.20%	4.20%	5.36%	4.90%	6.27%	TSP
Escherichia coli	10.10%	8.81%	11.25%	10.53%	13.47%	
Salmonella enterica	10.40%	8.71%	11.12%	1.29%	1.65%	
Lactobacillus fermentum	18.40%	10.60%	13.53%	0.00%	0.00%	
Enterococcus faecalis	9.90%	6.69%	8.54%	7.72%	9.88%	
Staphylococcus aureus	15.50%	11.24%	14.35%	12.61%	16.13%	
Listeria monocytogenes	14.10%	10.42%	13.31%	13.61%	17.41%	
Bacillus subtilis	17.40%	16.33%	20.85%	20.23%	25.87%	

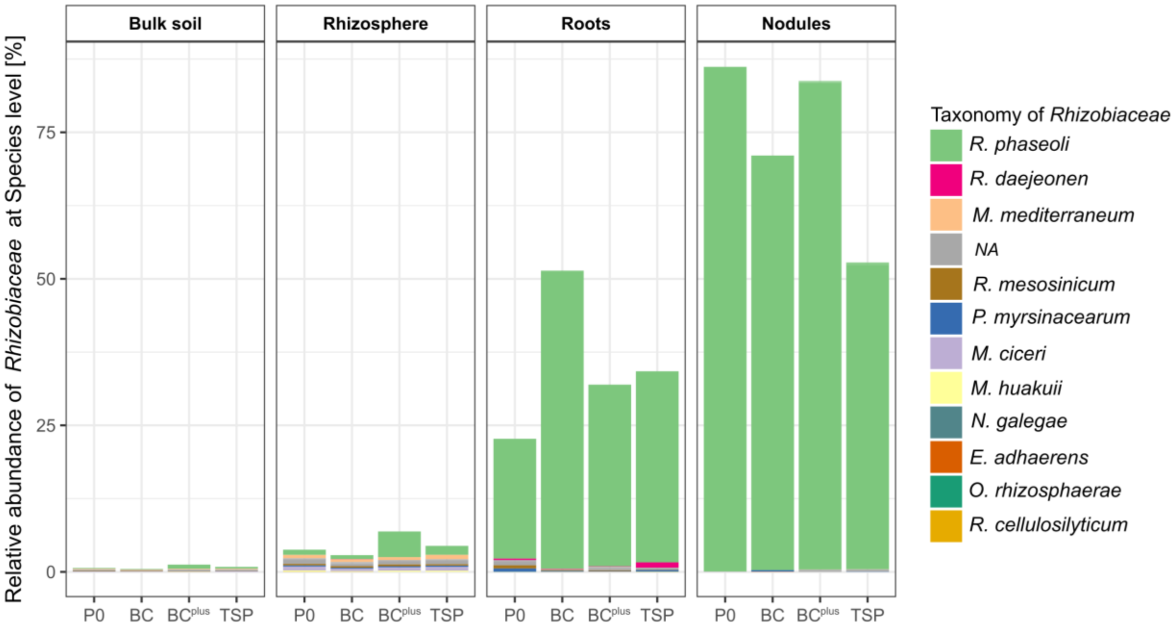

Figure S1 Relative abundance (%) of *Rhizobiaceae* at the species level in the different compartments (Bulk soil, Rhizosphere, Roots, Nodules) per fertilization treatment (P0, BC, BC^{plus}, TSP).

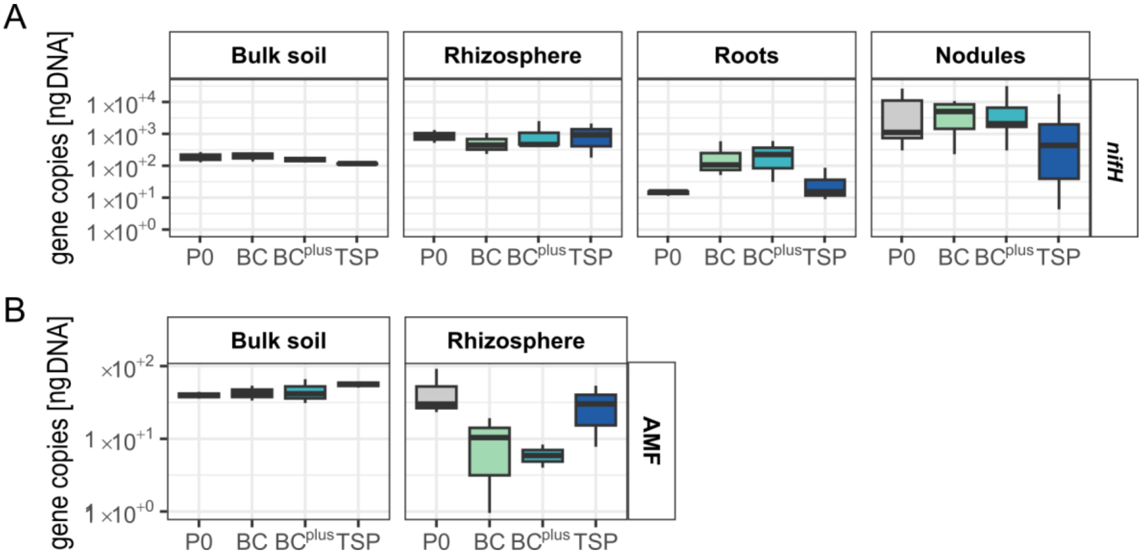

Figure S2 Quantification of gene copy numbers using qPCR. (A) Gene copies of *nifH* in the bulk soil, rhizosphere, roots, and nodules per µL DNA for the fertilization treatments P0, BC, BC^{plus,} and TSP plotted on a linear scale as box plot (n = 3 for rhizosphere and bulk soil, n = 9 for roots and nodules). (B) Gene copies of AMF in the bulk soil and rhizosphere per ng DNA for the fertilization treatments (P0, BC, BC^{plus,} and TSP) plotted on a linear scale as a box plot (n = 3). No significant differences were observed between the treatments.

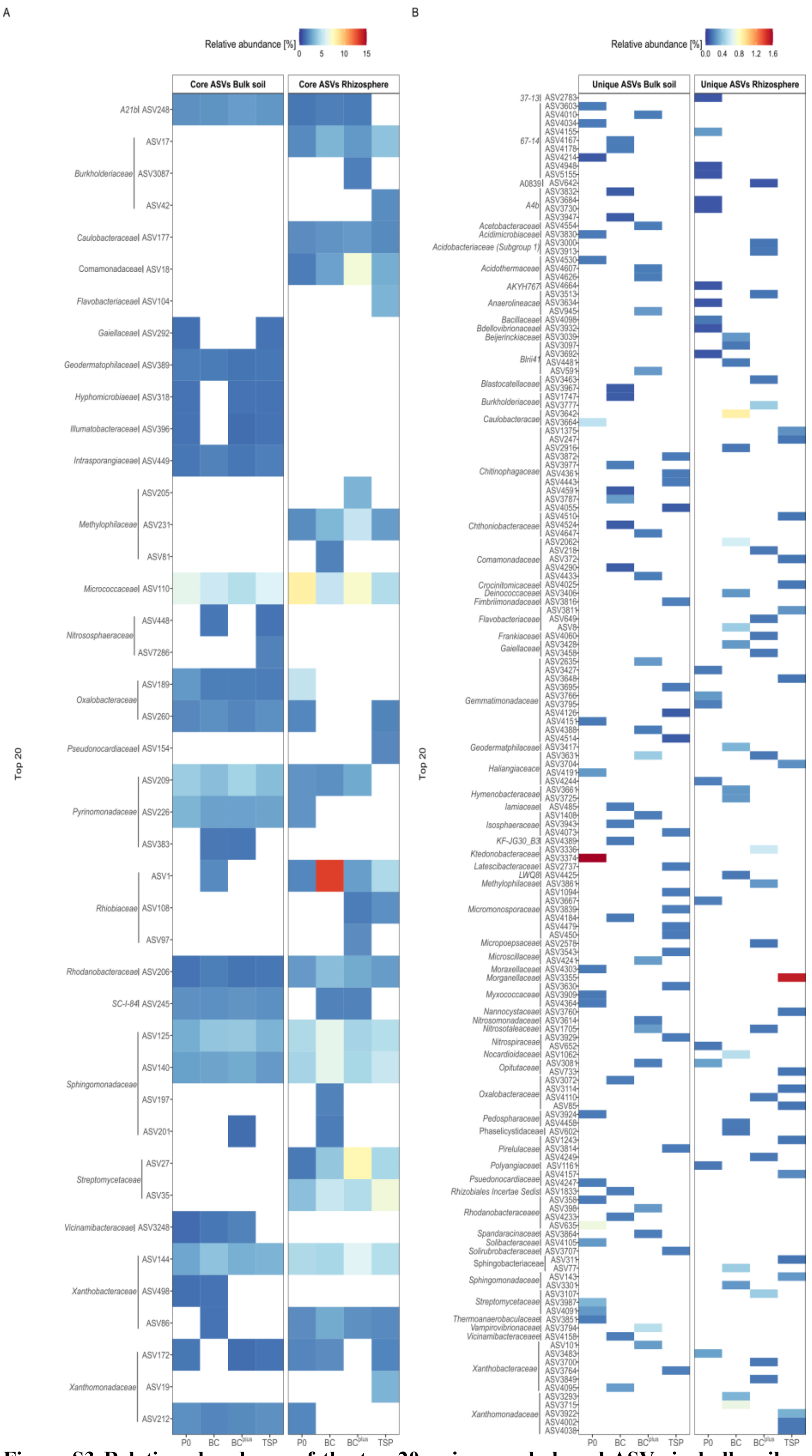

Figure S3 Relative abundance of the top 20 unique and shared ASVs in bulk soil and rhizosphere under phosphorus treatments. Heatmaps showing the relative abundance of the top 20 shared and unique ASVs at the family level in bulk soil and rhizosphere across phosphorus fertilization treatments (P0, BC, BC^{plus}, TSP) in bulk soil and rhizosphere. (A) Shared ASVs detected across all treatments.(B) Unique ASVs specific to individual treatments Darker shades indicate higher abundance.

Table S4 Primer, reaction mixture, conditions, and calibration standards for real-time qPCR.

Target gene	F- and R-primer (pmol µl^{-1})	Thermal profile	Primer name	Sequence 5‘-3‘	Fragment length (bp)	Reference	Calibration standard source
16S rRNA gene	0.5	95°C/45 sec, 58 °C/45 sec, 72 °C/45 sec	FP 16S<br>RP 16S	GGTAGTCYAYGCMSTAAACG<br>GACARCCATGCASCACCTG	264	(1)	*Pseudomonas putida*
LSU_AMF	0.5	94°C/30 sec, 60 °C/40 sec, 72 °C/60sec	FLR3<br>FLR4	TTGAAAGGGAAACGATTGAAGT<br>TACGTCAACATCCTTAACGAA	380	(2)	*Rhizophagus irregularis*
nifH	0.5	95°C/45 sec, 60 °C/45 sec, 72 °C/45 sec	nifH-f-Rosch<br>nifH-r-Rosch	AAAGGYGGWATCGGYAARTCCACCAC<br>TTGTTSGCSGCRTACATSGCCATCAT	458	(3)	*Sinorhizobium meliloti 30136*

Table S5 Read loss per processing step during the bioinformatic pipeline. The number after the letter in the Sample column indicates the respective plot from which the sample comes. B stands for bulk soil, R for rhizosphere, K for nodule, and W for root. The samples that were resequenced from the nodules and roots are labeled WDH.

Sample	filter+trim reads.in	filter+trim reads. out	dadaF	dadaR	merged	seqtable	removeBimera
B3	141154	133746	127902	128014	104232	104232	103519
B5	75973	72097	67543	68041	51352	51352	51066
B10	93616	88520	83880	84358	63726	63726	63377
B12	77652	74157	69431	70023	53096	53096	52780
B17	147995	140989	134691	135494	108806	108806	107839
B20	62060	58843	54411	55175	39874	39874	39808
B24	89383	84629	80030	80405	62039	62039	61697
B27	73630	69929	65058	65863	47426	47426	47327
B29	119088	111878	106739	107237	79684	79684	79005
B32	105362	100187	94828	95317	73237	73237	72667
B36	105421	99935	94788	95568	72171	72171	71512
B39	80677	76996	72309	72601	54265	54265	53963
R3	124976	119689	114867	115445	95870	95870	94835
R5	66782	64261	61136	61686	51048	51048	50483
R10	76894	73135	69093	69471	52944	52944	52617
R12	84638	80490	76326	76967	61047	61047	60768
R17	138517	131072	126249	127060	108141	108141	107348
R20	85966	81965	78174	78381	62446	62446	61974
R24	62783	56875	53233	53845	40999	40999	40805
R27	77718	74553	71055	71099	56915	56915	55966
R29	131675	125111	120404	120848	98790	98790	97908
R32	73778	70390	66375	66982	50407	50407	50094
R36	55829	52502	49476	50223	38766	38766	38255
R39	82194	78405	74380	75310	60153	60153	59691
C3 (Ex.kont)	6504	6092	5574	5603	4118	4118	4118
C5 (Ex.kont)	5770	5430	4908	4940	3439	3439	3426
Cw (Ex.kont)	8623	8208	7600	7762	6003	6003	5972
NN1 (PCR Kont.)	11426	10898	10339	10385	8135	8135	8103
NN2 (PCR Kont.)	9925	9412	8846	8746	6622	6622	6515
K3.1	103435	96029	95389	95386	94072	94072	93878
K3.2	143039	135160	134879	134863	134581	134581	134533
K3.3	147463	136356	136054	135948	135159	135159	135132
K5.1	256469	240691	240236	240250	239624	239624	239264
K5.2	268547	251559	250976	250943	247712	247712	246764
K5.3	212495	194026	193657	193703	191924	191924	191822
K10.1	220844	207827	206664	206638	204081	204081	203342
K10.2	167505	154942	154650	154591	153907	153907	153791
K10.3	118074	110336	110086	109984	109640	109640	108856
K12.1	232442	215668	214629	214710	208080	208080	206893
K12.2	264387	246730	246528	246399	246052	246052	245626

K12.3	152951	141199	140899	140749	140329	140329	139764
K17.1	303522	284162	283439	283364	279500	279500	273442
K17.2	154918	143672	143096	142636	133356	133356	133072
K17.3	52879	49601	49297	49325	48121	48121	47602
K20.1	194295	182432	182113	182007	181358	181358	180963
K20.2	133357	125813	125374	125357	123924	123924	122050
K20.3	177102	167471	167014	167215	163685	163685	163347
K24.1	118475	111982	111808	111754	111550	111550	111213
K24.2	99927	94366	94110	94099	93597	93597	93448
K24.3	137381	130155	129830	129814	129090	129090	128694
K27.1	106129	97400	97174	97174	96524	96524	96502
K27.2	75883	70934	70610	70673	70268	70268	70133
K27.3	81070	76724	76412	76455	76169	76169	75767
K29.1	89912	85485	85296	85211	85042	85042	84986
K29.2	47885	44428	44110	44141	43598	43598	43523
K29.3	63499	59990	59805	59763	59239	59239	59103
K32.1	47045	44369	44246	44265	43929	43929	42567
K32.2	74274	70359	70225	70186	69689	69689	67845
K32.3	76831	72288	72098	72135	71610	71610	69518
K36.1	77099	73580	73351	73415	71608	71608	71565
K36.2	68441	65099	64998	64978	64762	64762	64762
K36.3	210093	199028	198497	198616	197162	197162	196752
K39.1	34782	32264	31943	31910	29699	29699	29634
K39.2	61898	58493	58058	58216	57040	57040	56049
K39.3	62550	58815	58320	58468	57406	57406	56286
CK1 (Ex. Kont)	6915	5861	5341	5153	4140	4140	4140
CK2	1063	716	625	585	461	461	461
CK3	1426	933	865	821	744	744	744
CK4	1189	639	574	559	496	496	496
CK5	1070	518	438	415	363	363	363
CW	8821	8240	7726	7663	6128	6128	6056
NN1	1165	673	572	544	481	481	481
NN2	1861	1050	967	934	842	842	842
Pos 0	68075	64155	63874	63892	61197	61197	52698
Pos TSP	66063	62478	62150	62277	60026	60026	53048
Pos BC	58531	54770	54505	54556	52905	52905	47677
Pos BC^{plus}	62164	58903	58636	58758	57026	57026	51726
Pos PCR Neg	274	126	106	101	89	89	89
K3.1 WDH	173238	143892	143133	142884	133489	133489	133199
K3.2 WDH	134286	111527	111130	110872	104239	104239	104216
K17.3 WDH	63886	54810	54516	54510	51364	51364	50692
K27.2 WDH	110934	94160	93861	93760	88890	88890	88762
K29.2 WDH	81908	67477	67091	66995	63153	63153	63075

K29.3 WDH	83560	71487	71269	71138	67179	67179	66999
K32.1 WDH	104310	90178	89929	89837	85145	85145	82398
K36.3 WDH	176071	151176	150665	150702	142305	142305	142103
K39.1 WDH	50468	41664	41315	41364	38292	38292	38292
K39.2 WDH	74025	64235	63875	63861	60271	60271	59352
K39.3 WDH	67764	58278	57901	57930	54906	54906	53816
W3.1	42260	37126	36992	36968	36586	36586	36407
W3.2	54781	48294	48062	47990	47476	47476	46940
W3.3	26062	23208	23087	23087	22815	22815	22675
W5.1	64708	56268	56177	56139	55818	55818	55532
W5.2	61385	53283	53152	53096	52772	52772	52452
W5.3	45860	40188	40029	40052	39807	39807	39555
W10.1	53241	46177	46059	46048	45691	45691	45115
W10.2	41496	35826	35648	35641	35067	35067	34301
W10.3	31408	27593	27455	27460	27191	27191	26937
W12.1	70914	61319	61176	61163	60761	60761	60247
W12.2	92119	79887	79742	79734	79300	79300	78644
W12.3	85788	74882	74588	74619	73413	73413	70776
W17.1	53464	46718	46603	46565	46265	46265	46098
W17.2	72321	63612	63337	63402	62937	62937	62203
W17.3	52813	46550	46394	46380	45896	45896	45229
W20.1	89881	80630	79966	80015	77694	77694	76001
W20.2	43494	39052	38331	38484	36586	36586	34793
W20.3	44559	39850	39162	39230	37254	37254	35493
W24.1	51737	46258	46099	46044	45370	45370	44597
W24.2	31666	28259	28077	28068	27463	27463	26436
W24.3	24567	21859	21681	21723	21267	21267	20688
W27.1	49378	43758	43559	43569	42887	42887	42334
W27.2	63452	55744	55430	55419	54350	54350	51683
W27.3	35073	31149	30793	30891	30085	30085	28713
W29.1	43833	38078	37943	37876	37609	37609	37334
W29.2	27755	24221	24047	24005	23786	23786	23186
W29.3	22996	20272	20132	20148	19973	19973	19603
W32.1	44213	38892	38569	38554	37635	37635	37300
W32.2	22419	19571	19263	19269	18712	18712	18564
W32.3	33152	29193	28808	28777	28116	28116	27704
W36.1	76095	67753	67209	67222	65236	65236	64014
W36.2	31818	28184	27732	27768	26559	26559	25751
W36.3	53521	47693	47209	47195	45497	45497	44027
W39.1	85558	73998	73741	73749	72929	72929	72312
W39.2	49394	43068	42731	42803	41893	41893	41091
W39.3	71614	62544	62084	62130	60866	60866	59701
CW	4332	2427	2321	2407	2260	2260	2260

Pos 0	17053	15240	15015	15063	14001	14001	12634
Pos TSP	31947	28460	28149	28226	26504	26504	22576
Pos BC	42779	38232	37996	37968	36559	36559	34499
Pos BC^{plus}	33748	30355	29933	29953	28141	28141	25215
NN1	6960	4856	4780	4833	4767	4767	4670
NN2	8142	5633	5553	5592	5441	5441	5441
W3.3 WDH	53343	46419	46150	46164	45388	45388	44980
W5.1 WDH	130768	112308	112091	112114	110109	110109	108778
W5.2 WDH	135258	115981	115727	115693	113965	113965	112464
W5.3 WDH	109141	93795	93520	93617	92193	92193	90690
W10.1 WDH	119213	102614	102364	102401	100922	100922	99270
W10.2 WDH	86805	74473	74158	74177	72374	72374	69615
W10.3 WDH	70977	61303	61003	61067	59984	59984	58807
W12.1 WDH	153345	132183	131855	131969	129252	129252	127208
W12.2 WDH	202192	172529	172163	172227	169541	169541	167182
W12.3 WDH	198955	172408	171897	172053	167819	167819	159687
W17.1 WDH	107788	92684	92485	92449	91159	91159	90464
W17.3 WDH	126667	109554	109195	109288	106649	106649	104273
W27.3 WDH	72781	63414	62856	63061	61042	61042	57066
W29.1 WDH	78894	68243	67762	67759	66219	66219	65063
W29.2 WDH	62465	53923	53619	53647	52599	52599	50472
W29.3 WDH	59116	51244	51001	51051	50038	50038	48876
W32.1 WDH	16	1	1	1	0	0	0
W32.2 WDH	38678	33366	32891	32965	31982	31982	31375
W32.3 WDH	79122	68373	67740	67817	65888	65888	63977
W39.2 WDH	99346	85219	84621	84725	82626	82626	79716
W39.3 WDH	162486	140955	140213	140324	136513	136513	132083

References:

1. Bach H-J, Hartmann A, Schloter M, Munch J. PCR primers and functional probes for amplification and detection of bacterial genes for extracellular peptidases in single strains and in soil. J Microbiol Methods. 2001;44(2):173-82.
2. Gollotte A, Van Tuinen D, Atkinson D. Diversity of arbuscular mycorrhizal fungi colonising roots of the grass species Agrostis capillaris and Lolium perenne in a field experiment. Mycorrhiza. 2004;14(2):111-7.
3. Rösch C, Mergel A, Bothe H. Biodiversity of denitrifying and dinitrogen-fixing bacteria in an acid forest soil. *Appl Environ Microbiol*.. 2002;68(8):3818-29.
